# Treatment of recalcitrant lupus erythematosus tumidus with deucravacitinib

**DOI:** 10.1016/j.jdcr.2023.11.029

**Published:** 2023-12-16

**Authors:** Arianna Zhang, Rebecca G. Gaffney, Joseph F. Merola

**Affiliations:** a School of Medicine, Tufts University, Boston, Massachusetts; b Department of Dermatology, Brigham and Women’s Hospital, Harvard Medical School, Boston, Massachusetts; c Department of Dermatology, UT Southwestern Medical Center, Dallas, Texas; d Division of Rheumatology, Department of Medicine, UT Southwestern Medical Center, Dallas, Texas

**Keywords:** autoimmune disease, cutaneous lupus erythematosus, deuravacitinib, treatment, Tyk2

## Introduction

Cutaneous lupus erythematosus (CLE) is a potentially scarring and disfiguring condition, often recalcitrant to first-line therapy, for which no treatments have been approved by the Food and Drug Administration. Deucravacitinib, a tyrosine kinase 2 inhibitor which has been approved for treatment of psoriasis, has downstream effects on type I interferon signaling and is a promising therapy for treatment of cutaneous lupus. Herein, we report a case of tumid lupus erythematosus (TLE) that was recalcitrant to multiple therapies, but ultimately treated successfully with deucravacitinib.

## Case report

**Fig 1:**
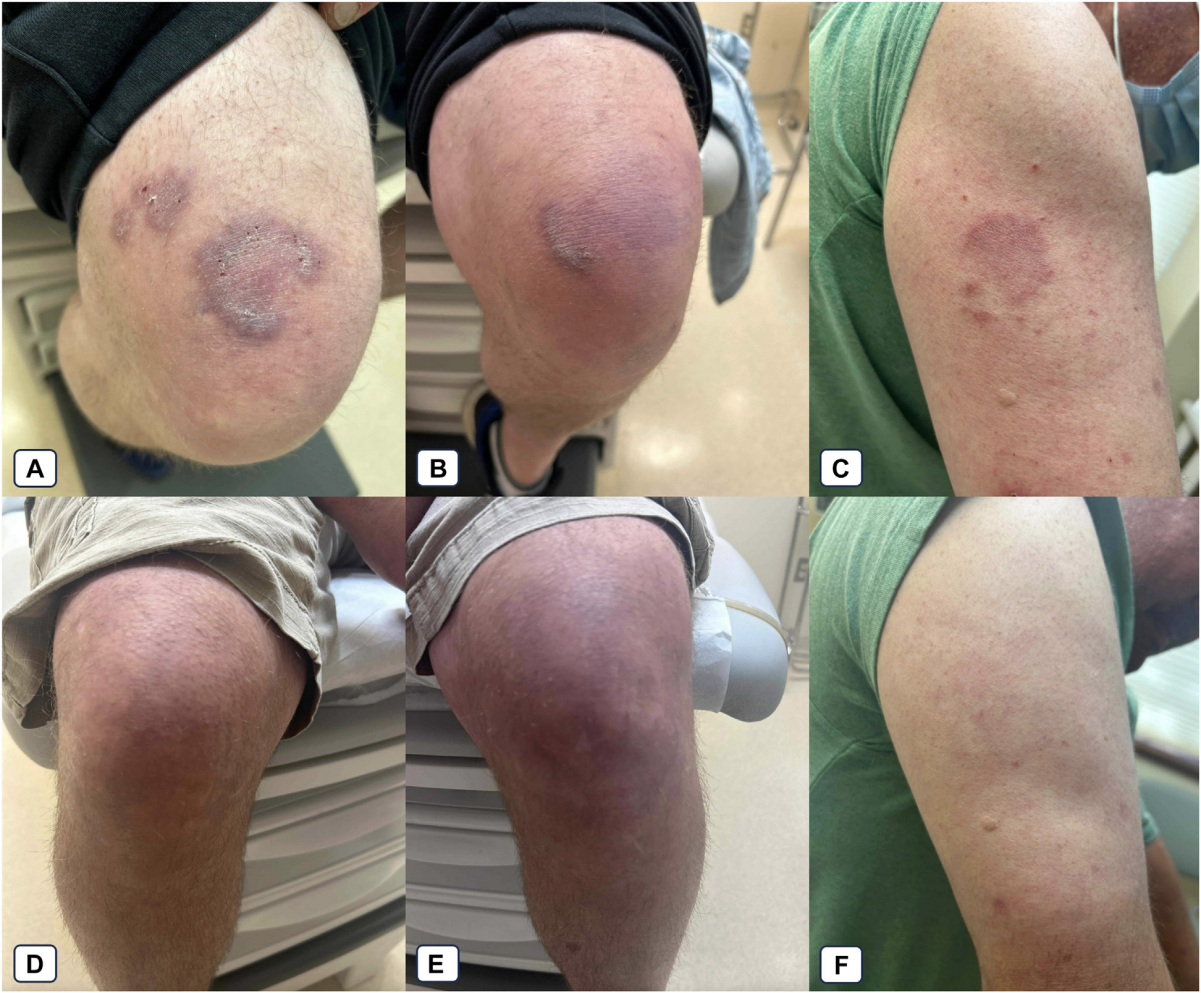
A 51-year-old male pretreatment and posttreatment of tumid lupus erythematosus with deucravacitinib after 4 months. **A**, Right anterior lower extremity, **B**, left anterior lower extremity, and **C**, right upper extremity with violaceous, indurated plaques before addition of deucravacitinib therapy. **D**, Right anterior lower extremity, **E**, left anterior lower extremity, and **F**, right upper extremity 3 months after initiation of deucravacitinib therapy, with residual improved violaceous changes and minimal skin thickening at sites of previous plaques.

A 51-year-old male active smoker with a family history of systemic lupus erythematosus (SLE) was referred to our outpatient clinic for a recurrent, photosensitive, cutaneous eruption present over several years. The rash was previously biopsied by an outside provider and consistent with TLE. The patient also reported diffuse joint pain. On physical examination, he was found to have erythematous, edematous papules, and plaques in photodistributed areas (helices, face, extensor forearms, and dorsal aspect of the hands), as well as knees and abdomen. A subset of these, particularly on the lower extremities, was annular with central hyperpigmentation and raised borders (Fig 1, *A-C*). Rheumatologist joint examination did not reveal synovitis or inflammatory arthritis. The differential diagnosis included other cutaneous lupus variants including discoid lupus, chilblains, granulomatous reaction, or atypical infection; ultimately however, TLE was favored in the context of the biopsy and morphology, smoking history, and photoexacerbation. Systemic autoimmune disease workup, including antinuclear antibodies, double-stranded deoxyribonucleic acid, C3/C4, and rheumatoid factor, was negative. Repeat punch biopsies of lesions on the right knee and right forearm both demonstrated prominent papillary dermal edema and “cuffed” perivascular and periadnexal lymphocytic infiltrates and mucin deposition, without interface dermatitis consistent with TLE. Over the course of several years, the patient failed or had an incomplete response to numerous therapies including: high potency topical corticosteroids, topical tacrolimus, oral corticosteroids, hydroxychloroquine, chloroquine, methotrexate, pentoxifylline, mycophenolate mofetil, and thalidomide. The patient also modified lifestyle behaviors such as photoprotection and smoking reduction. Given his treatment-refractory disease, the patient was started on deucravacitinib, an oral tyrosine kinase 2 inhibitor, while continuing baseline chloroquine 250 mg daily. Deucravacitinib was dosed at 6 mg daily, consistent with the Food and Drug Administration-approved psoriasis dosing regimen. At his 3-month follow-up, the patient reported noticeable improvement of his symptoms and rash, with minimal induration and only minimal violaceous changes remaining (Fig 1, *D-F*). He tolerated the medication well with no side effects and was continued on this regimen.

## Discussion

Tumid lupus erythematosus is an uncommon, highly photosensitive variant of chronic CLE that is less commonly associated with SLE. Although no drugs have yet to be approved for CLE, several newer agents including deucravacitinib have demonstrated good clinical efficacy for skin symptoms in SLE trials.^1^^,^^2^ A subset of early phase II clinical trial data demonstrated that deucravacitinib achieved significant reduction in cutaneous lupus symptoms compared with placebo (Cutaneous Lupus Erythematosus Disease Area and Severity Index-50 response 69.6% versus 16.7%; *P* < .001).^2^ The mechanism of this therapeutic effect is theorized to relate to tyrosine kinase 2 inhibitors’ downstream effect on type I interferon signaling.

Deucravacitinib may be considered as a second- or third-line currently off-label treatment option for CLE recalcitrant to other first- and second-line therapies; its overall favorable safety and tolerability profile make it a particularly compelling option for second-line use in CLE. Upcoming skin efficacy data from phase III clinical trials of deucravacitinib in SLE may further support its use for the treatment of cutaneous lupus symptoms. This case is presented not only to demonstrate successful use of deucravacitinib in a patient with skin-limited lupus, but also to highlight the unmet need for evidence-based targeted treatment options for patients with CLE.

## Conflicts of interest

Dr Merola is a consultant and/or investigator for Amgen, Astra Zeneca, Boehringer Ingelheim, Bristol-Myers Squibb, AbbVie, Dermavant, Eli Lilly, Incyte, Novartis, Janssen, UCB, Sanofi-Regeneron, Sun Pharma, Biogen, Pfizer, and Leo Pharma. Author Zhang and Dr Gaffney have no conflicts of interest to declare.
